# Cell cycle arrest and apoptosis induction by *Juniperus communis* extract in esophageal squamous cell carcinoma through activation of p53‐induced apoptosis pathway

**DOI:** 10.1002/fsn3.2084

**Published:** 2020-12-30

**Authors:** Chia‐Yu Li, Shan‐Chih Lee, Wen‐Lin Lai, Kai‐Fu Chang, Xiao‐Fan Huang, Peng‐Yun Hung, Chi‐Pin Lee, Ming‐Chang Hsieh, Nu‐Man Tsai

**Affiliations:** ^1^ Department of Life‐and‐Death Studies Nanhua University Chiayi Taiwan; ^2^ Department of Medical Imaging and Radiological Sciences Chung Shan Medical University Taichung Taiwan; ^3^ Department of Medical Imaging Chung Shan Medical University Hospital Taichung Taiwan; ^4^ Department of Medical Laboratory and Biotechnology Chung Shan Medical University Taichung Taiwan; ^5^ Clinical Laboratory Chung Shan Medical University Hospital Taichung Taiwan; ^6^ Institute of Medicine Chung Shan Medical University Taichung Taiwan; ^7^ Division of Cardiology Department of Internal Medicine Distmanson Medical Foundation Chia‐Yi Christian Hospital Chia‐Yi Taiwan

**Keywords:** apoptosis, cell cycle, esophageal squamous cell carcinoma, *Juniperus communis*, synergistic effect

## Abstract

Esophageal squamous cell carcinoma (ESCC) is one of the most common cancers. It has a high mortality rate and requires novel effective drugs and therapeutic approaches. *Juniperus communis* (JCo), used to flavor gin and food, has been documented to have anti‐tumor activity. The aim of this study was to investigate the antitumor activity of JCo extract against ESCC and its possible mechanisms. JCo extract suppressed cell growth in ESCC and showed higher selection for ESCC cells than normal cells compared to the clinical drug 5‐fluorouracil (5‐FU). JCo extract induced cell cycle arrest at the G_0_/G_1_ phase by regulating the expression of p53/p21 and CDKs/cyclins, triggering cell apoptosis by activating both the extrinsic (Fas/FasL/Caspase 8) and intrinsic (Bcl‐2/Bax/Caspase 9) apoptosis pathways. Moreover, a combination treatment of JCo and 5‐FU synergistically inhibited proliferation of ESCC cells. These results suggest that JCo extract is a potential natural therapeutic agent for esophageal cancer, as it could induce cell cycle arrest and apoptosis in ESCC cells.

## Graphical abstract

Extract of Juniperus communis inhibits the growth of esophageal squamous cell carcinoma cells by stimulating G0/G1 arrest and inducing cell apoptosis via regulation of relative proteins. Moreover, JCo extract enhances the anticancer effect of the clinical drug 5‐FU, suggesting that it can complement current chemotherapeutic treatment.

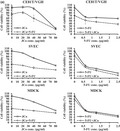

## 1 INTRODUCTION

Human esophageal carcinoma is one of the most frequently diagnosed cancers, and the sixth leading cause of cancer‐related deaths worldwide, causing the deaths of over 400,000 people annually (Pennathur et al., 2013; Siegel et al., 2016). Esophagus squamous cell carcinoma (ESCC) is an aggressive cancer and the most common histological type seen in China, South America, and Western Europe (Chen et al., 2016; Siegel et al., 2016). In recent years, the overall 5‐year survival rate for late‐stage ESCC has been less than 15%, and the prognosis remains poor for patients, although the development of therapeutic approaches, such as surgery, chemotherapy and radiotherapy (Liang et al., 2017; Wu et al., 2013; Wu et al., 2012). 5‐fluorouracil (5‐FU) has been the first‐line treatment for ESCC therapy for several decades and is also widely used in the treatment of head and neck carcinomas (Longley et al., 2003). However, single agent chemotherapy with 5‐FU is no longer appropriate for ESCC therapy because of its toxicity, drug resistance, and side effects with increasing doses. Therefore, combination therapy with other drugs, such as irinotecan and oxaliplatin, is used to improve prognoses and reduce the side effects of 5‐FU (Ferte et al., 2011; Wainberg et al., 2011). Combination therapy improves the response rate and survival rate, but the side effects and drug resistance are still unknown.

Natural products and natural health products (extracts or essential oils) from plants are complex mixtures containing various organic compounds that are important for the development of new drugs for anticancer, antibacterial, anti‐inflammatory, and antioxidant purposes (Mann, 2002; Padumadasa et al., 2016; Rong et al., 2016; Soukand et al., 2015). In clinical cancer therapy, there are at least 73 approved anticancer drugs from plants, such as topotecan, etoposide, and paclitaxel (Lin et al., 2015; Pan et al., 2010). Moreover, these natural products can also be used in combination with chemotherapy or radiotherapy, resulting in an enhanced therapeutic efficacy and decreased side effects in cancer treatments (Mileo & Miccadei, 2016). Therefore, an increasing number of researchers are now focusing on investigations of the anti‐cancer potential of plants.

*Juniperus communis* (JCo) is a coniferous evergreen shrub, used to flavor gin and food, widely distributed throughout the Northern Hemisphere and across the Himalayas from Kumaun region at an altitude of 1,700–4,200 m (Khare, 2007; Moein et al., 2010; Nakanishi et al., 2004). *Juniperus communis* oil contains monoterpene hydrocarbons, such as α‐pinene, β‐pinene myrcene, and sabinene (Bais et al., 2014; Cabral et al., 2012; Hajdari et al., 2015). In traditional medicine, *Juniperus* plants are widely used to relieve colds, headaches, respiratory diseases, asthma, and digestive and gynecological disorders (Leporatti & Ivancheva, 2003). Recent studies report that *Juniperus* (essential oils or extracts) shows antioxidant, anti‐microbial, anti‐inflammatory, nephroprotective, and hepatoprotective effects (Al‐Attar, Alrobai, & Almalki, 2016, 2017; Carpenter et al., 2012; Orhan et al., 2011). Furthermore, the cytotoxic effects of *Juniperus* species have been investigated in different cancer types, including melanoma, neuroblastoma, leukemia, lung, breast, and colon cancers (Bayazit, 2004; Gao et al., 2019; Lantto et al., 2016; Pollio et al., 2016; Yaman et al., 2019). In this study, we evaluated the anti‐cancer potential of JCo extract by investigating its effects on anti‐proliferation, the cell cycle, and apoptosis in the human esophageal squamous cell carcinoma cell line CE81T/VGH.

## 2 MATERIALS AND METHODS

### 2.1 Cell line culture conditions and reagents

CE81T/VGH (human esophageal squamous cell carcinoma), CE48T/VGH (human esophageal epidermoid carcinoma (VGH), SVEC (mouse vascular endothelial cell) and MDCK (cannis kidney epithelial cells) were purchased from the Food Industry Research and Development Institute. CE81T/VGH, CE48T/VGH, SVEC, and MDCK were cultured in Dulbecco's Modified Eagle Medium supplemented with 10% fetal bovine serum (Gibco BRL), 10 mM HEPES (Gibco), 1 mM pyruvate (Gibco), P/S (100 μg/ml penicillin, and 100 μg/ml streptomycin; Gibco), and non‐essential amino acids (Gibco, CE81T/VGH and CE48T/VGH only). Cells were grown in 10 cm^2^ culture dishes in a humidified atmosphere with 5% CO_2_ at 37°C. The TP53 in the CE81T/VGH cells was mutated, which was detected using automated extraction of nucleic acids (AccuBioMed Co., Ltd.) and Femtopath Human Primer Sets (HongJing Biotech). JCo was purchased from the PHOENIX company and extracted using steam distillation. The clinical drug 5‐Fluorouracil (5‐FU; Sigma) was prepared in dimethyl sulfoxide (DMSO) in each in vitro experiment. Cells were treated with equivalent amounts of DMSO in the control and treatment groups, and the final concentration of DMSO in each experiment was 0.01%–0.5%.

### 2.2 Cell viability and proliferation assay

The viability of CE81T/VGH, CE48T/VGH, MDCK, and SVEC cells was determined using a modified 3‐(4,5‐dimethylthiazol‐2‐yl)‐2,5‐diphenyltetrazolium bromide (MTT) assay. Cells were cultured in a 96‐well plate at a density of 5 × 10^3^ cells/well for 24 hr and treated with different concentrations of JCo extract or 5‐FU for 24, 48, and 72 hr. MTT was dissolved in the base medium (400 μg/mL, Sigma), and 100 μl of solution was added into each well and incubated for 6–8 hr. The MTT formazan crystals were dissolved in 50 μL DMSO and the optical density (O.D.) was detected using a microplate reader (Molecular Devices, Spec384) at 550 nm. Cell viability was calculated as the O.D. percentage relative to the controls. The half‐maximal inhibitory concentration (IC_50_) value of JCo extract was measured from the cell viability assay.

### 2.3 Flow cytometric cell cycle analysis

The effect of JCo extract on the cell cycle distribution of CE81T/VGH was determined by flow cytometry. Cells were seeded at a density of 2 × 10^6^ per 100 mm culture dish, incubated overnight, and treated with 70 μg/ml JCo extract for 0, 6, 12, 24, or 48 hr for time course analysis; for dosage analysis, cells were treated with 0, 50, 70, or 90 μg/ml JCo extract for 24 hr. Cells were harvested with trypsin‐EDTA, washed with PBS, and incubated in 40 μg/ml propidium iodide (Sigma) solution containing 0.1 mg/ml RNase A (Sigma) at 4°C overnight. The DNA content was then detected using FACScan (Beckton Dickinson) and analyzed with Kaluza Flow Cytometry Analysis Software (Software Version 1.2, Beckman Coulter).

### 2.4 TUNEL assay

Apoptosis was detected using the Situ Cell Death Detection Kit (Roche, Mannheim, Germany). Cells were treated with 70 μg/ml JCo extract for 48 hr, collected, and fixed with 10% formaldehyde for 10 min. The cells were smeared and dried on silane‐coated glass slides and rehydrated with PBS. After using 3% H_2_O_2_ in methanol to inactivate endogenous peroxidase for 10 min, the cells were permeabilized with 0.1% Triton X‐100 in 0.1% sodium citrate on ice for 2 min. Cells were incubated with TUNEL solution for 2 hr at 37°C and stained with PI. The TUNEL‐positive cells showed green fluorescence, and cell images were taken using a fluorescence microscope (ZEISS AXioskop2) at 400× magnification.

### 2.5 Western blot analysis

CE81T/VHG cells were seeded in 100 mm culture dishes for incubation overnight, and then treated with 70 μg/mL JCo extract for 0, 6, 12, 24, or 48 hr. Cells were lysed with ice‐cold RIPA buffer containing protease inhibitor (BIO BASIC INC.) and phosphatase inhibitor (Bionovas) and incubated at 4°C for 30 min. The resulting lysates were centrifuged at 12,000 × *g* for 30 min at 4°C, and the protein concentration was determined using a bicinchoninic acid protein assay kit (Pierce, Rockford, IL, USA). Cell extracts (20 μg protein/lane) were separated using 8%–12.5% SDS‐PAGE gel electrophoresis and transferred to polyvinylidene difluoride membranes (FluoroTrans, PALL). The membrane was blocked with 10% skim milk and incubated with primary antibodies or β‐actin (iReal Biotechnology) in 1% Bovine serum albumin (in 1× TBS with 0.1% tween‐20) overnight, followed by incubation with biotin conjugated secondary antibodies for 2 hr. Finally, membranes were incubated with horseradish peroxidase‐conjugated streptavidin (Dianova) for 1 hr and proteins were detected using an ECL kit (T‐Pro Biotechnology) and chemiluminescence imaging analyzer (GE LAS‐4000, GE healthcare Life. Sciences). Protein bands were quantified using the software ImageJ 1.47t and normalized to the mean values of the untreated control and β‐actin.

### 2.6 Caspase activation analysis

CE81T/VGH cells were seeded at a density of 5 × 10^5^ in 6 well culture plates and incubated overnight. After pretreatment with 1 μM Z‐DEVD‐FMK (Biosciences, USA) for 2 hr, cells were treated with 70 μg/ml JCo extract for 24 hr. Cells were harvested and the protein level of pro‐caspase‐3 were detected by western blotting.

### 2.7 Immunocytochemistry staining

Cells were cultured in 100 mm culture dishes overnight and treated with JCo extract for 24 hr. The treated cells were collected, fixed with 4% paraformaldehyde, smeared, and dried on silane‐coated glass slides. Endogenous peroxidase was inactivated via treatment with 3% H_2_O_2_ for 10 min, and 10% BSA in PBS was used to block nonspecific binding. The target proteins were detected by immunostaining with anti‐p‐p53, anti‐p‐RB, anti‐Caspase 3, anti‐Caspase 8, and anti‐Caspase 9 (1/200 dilution) and anti‐p21 (iReal Biotechnology Co., Ltd.), and visualized with the HRP‐DAB detection system (HK542‐XAK, BioGenex).

### 2.8 Drug combination effect

Cells were seeded in 96 well culture plates (5 × 10^3^ cells/well) or 100 mm culture dishes (2 × 10^6^ cells/dish) for 24 hr, and treated with a combination of JCo extract (0, 20, 40, and 80 μg/ml) and/or 0.5 μg/ml 5‐FU; 5‐FU (0, 0.625, 1.25, and 2.5 μg/ml) and/or 30 μg/ml JCo extract for 48 hr. The cell viability of treated cells was measured using an MTT assay. The combination effect of the drug‐drug interaction was determined based on combination index (CI) and normalized isobologram using CompuSyn software (ComboSyn, Inc., Paramus, NJ, USA).

### 2.9 Statistical Analysis

The data are presented as the mean ± standard deviation (*SD*). One‐way ANOVA or Student's *t*‐test were used to analyze the differences between each group, and *p* values <.05 were considered statistically significant. All experiments were repeated at least 3 times in duplicates or triplicates.

## 3 RESULTS

### 3.1 JCo extract inhibited growth and proliferation of esophageal cancer cells but not normal cells

The major components of JCo extract were analyzed by using gas chromatography‐mass spectrometry (GC‐MS), including α‐pinene (34.87%), citronellyl acetate (14.26%), limonene (10.72%), trpinolene (10.65%), p‐cymene (6.21%), elemene (3.32%) and cadinene (2.12%), determined in our recent study (Gao et al., 2019).

**FIGURE 1:**
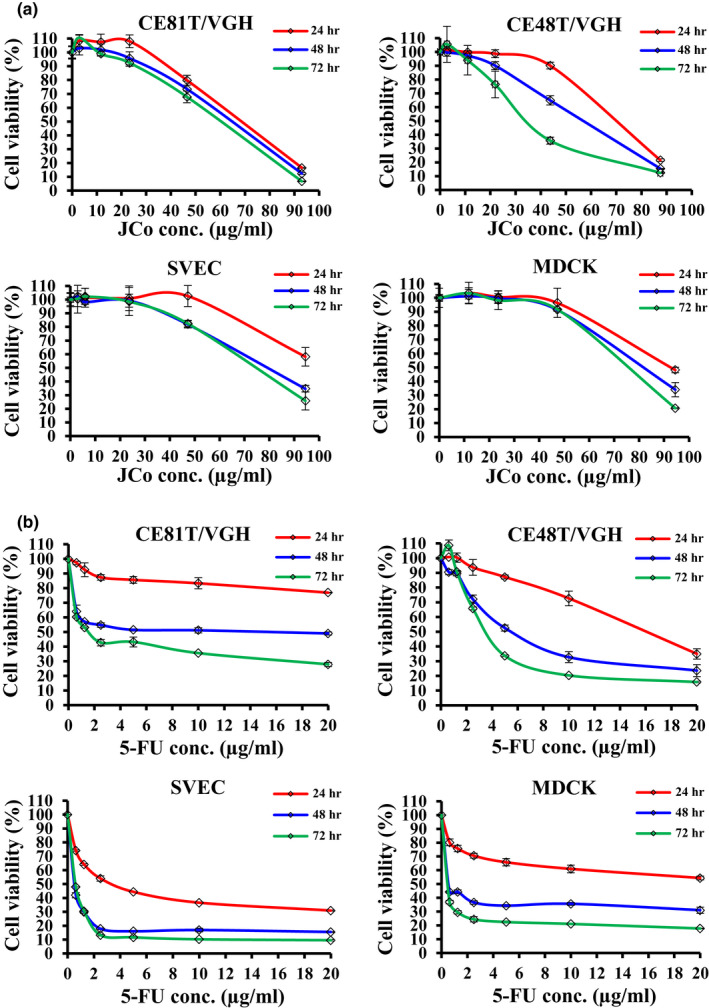
The inhibitory effect of JCo extract on the growth of esophageal cancer cells. CE81T/VGH, CE48T/VGH, SVEC, and MDCK cells were treated with (a) JCo extract and (b) 5‐FU at various concentrations for 24, 48, and 72 hr, followed by determination of cell viability by an MTT assay. The *P* value was calculated versus control cells (*p < .05). Data are presented as the mean ± *SD* of three independent experiments

To explore the growth‐inhibitory effect of JCo extract on esophageal cancer cells, cell viability was measured by an MTT assay. CE81T/VGH and CE48T/VGH cells, treated with different concentrations of JCo extract or 5‐FU, were significantly inhibited in a time‐ and dose‐dependent manner (Figure 1a,b). The estimated IC_50_ values of JCo extract after 24–72 hr treatment were 68.41 ± 1.38–60.07 ± 2.18 μg/ml in CE81T/VGH cells; 69.38 ± 0.95–36.10 ± 4.19 μg/ml in CE48T/VGH cells; >94.5–74.32 ± 3.45 μg/ml in SVEC cells; 92.68 ± 2.21–75.00 ± 1.53 μg/ml in MDCK cells (Table 1). These results show JCo extract treatment strongly inhibits the growth of esophageal cancer cells but has less effect in normal cells.

**TABLE 1:** The IC_50_ values of JCo extract against ESCC and normal cells

Cell lines	CE81T/VGH	CE48T/VGH	SVEC	MDCK
JCo				
24 hr	68.41 ± 1.38^b^	69.38 ± 0.95^a^, ^b^	>94.5	92.67 ± 2.21
48 hr	64.33 ± 2.56^a^, ^b^	56.96 ± 2.16^a^, ^b^	79.13 ± 2.09	81.32 ± 2.94
72 hr	60.07 ± 2.18^a^, ^b^	36.10 ± 4.19^a^, ^b^	74.32 ± 3.45	75.00 ± 1.53
5‐FU				
24 hr	>20	16.00 ± 0.03	3.53 ± 0.29	>20
48 hr	15.35 ± 6.74	5.64 ± 0.34	<0.63	<0.63
72 hr	1.62 ± 0.04	3.73 ± 0.06	<0.63	<0.63

Values are mean ± SD (μg/ml).

^a^ A significant difference between the JCo group compared with the 5‐FU group.

^b^ A significant difference between the ESCC cells compared with normal cells.

### 3.2 JCo extract induced G_0_/G_1_ phase arrest in CT81T/VGH cells

**FIGURE 2:**
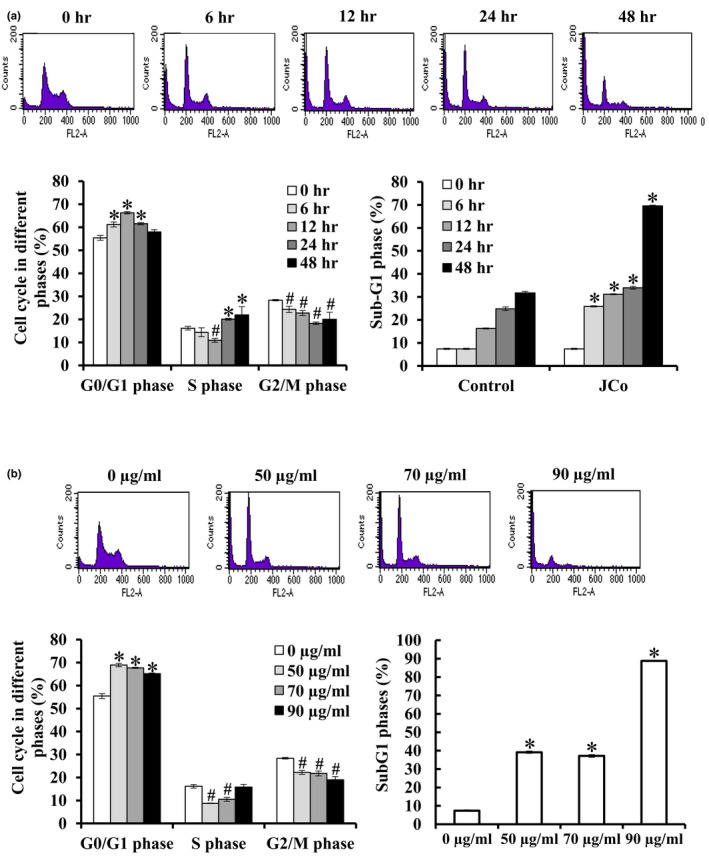
JCo extract induced G_0_/G_1_ phase arrest in esophageal cancer cells. Flow cytometry analysis of the DNA content of CE81T/VGH cells subjected to the JCo extract treatment for the indicated time (a) and dosage (b). Statistical analysis of cell subpopulations for each phase of the cell cycle was performed using the Kaluza Flow Cytometry Analysis Software. The percentage of the subG_1_ phase of JCo extract treated cells was significantly increased compared with the control, *p < .05; significantly decreased compared with control, #*p* < .05

To investigate the factors contributing to the growth inhibition of CE81T/VGH cells, we analyzed the effect of JCo extract on cell cycle distribution of a CE81T/VGH cell culture under different concentration of JCo extract (50, 70, and 90 μg/ml) for 0–48 hr. The flow cytometry results showed the average proportion of the G_0_/G_1_ phase after 1, 3, 6, 12, and 48 hr was 55.44 ± 1.06%, 61.21 ± 1.11%, 66.31 ± 0.47%, 61.56 ± 0.46% and 57.97 ± 1.00% after 70 μg/ml treatment, respectively (Figure 2a). For dosage analysis, the average proportion of the G_0_/G_1_ phase after treatment with 0, 50, 70, and 90 μg/ml of JCo extract for 24 hr was 55.44 ± 1.06%, 68.96 ± 0.76%, 67.72 ± 0.26% and 65.22 ± 0.23%, respectively (Figure 2b). Moreover, the percentage of cells in the subG_1_ phase increased in a time‐dependent and dose‐dependent manner after JCo extract treatment (Figure 2a,b). The results suggest that JCo extract blocks CE81T/VGH cells in the G_0_/G_1_ phase, triggers cell death, and increases the percentage of cells in the subG_1_ phase.

### 3.3 Effect of JCo extract on apoptosis detected by a TUNEL assay

**FIGURE 3:**
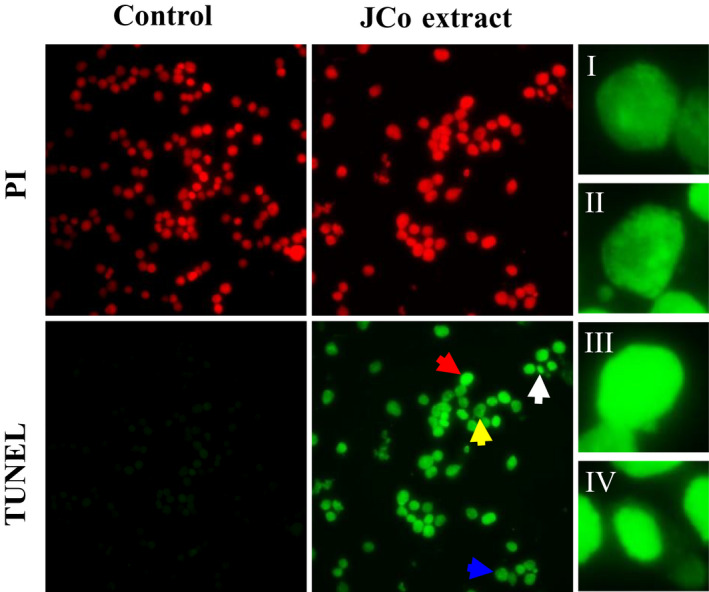
JCo extract induced cell apoptosis in esophageal cancer cells. CE81T/VGH cells were treated with 70 μg/mL JCo extract for 48 hr, and apoptosis was determined by a TUNEL assay. In the fluorescence images, anoikis (I, blue arrow), chromatin condensation (II, yellow arrow), DNA fragmentation (III, red arrow), and an apoptotic body (IV, white arrow) are shown

To determine the effect of JCo extract on apoptosis induction, apoptotic morphology was observed via the TUNEL assay kit. Cells showed TUNEL‐positive results after JCo extract treatment for 48 hr, and anoikis, chromatin condensation, DNA fragmentation, and apoptotic bodies were observed under a fluorescence microscope (Figure 3). The results indicate that JCo extract induces cell apoptosis in ESCC.

### 3.4 Anti‐cancer molecular mechanism of JCo extract in CE81T/VGH

**FIGURE 4:**
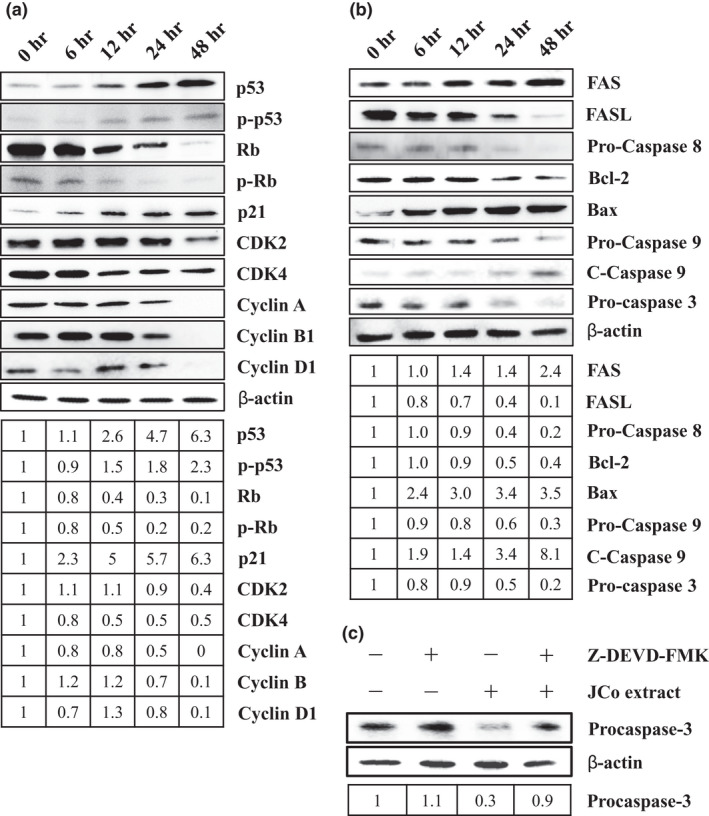
Cell cycle and apoptosis relative protein expression in JCo extract treated cells. CE81T/VGH cells were treated with 70 μg/ml of JCo extract for 6, 12, 24, or 48 hr, and western blotting was used to detect altered expression of the cell cycle (a) and apoptosis (b) relative proteins. (c) Cells pretreated with Z‐DEVD‐FMK, a caspase 3/7 inhibitor, for 2 hr, and then treated with 70 μg/ml of JCo extract for 24 hr. Protein expression of Pro‐Caspase 3 was detected by western blotting

Next, to examine the molecular mechanisms of the cell cycle in JCo extract treated cells, the expression levels of cell cycle regulatory proteins, including p53, Rb, p21, CDK2, CDK4, cyclin A, cyclin B1, and cyclin D1 were assessed by western blotting. p53, p‐p53, and p21 were increased, but Rb and p‐Rb were decreased, indicating that the cell cycle was regulated by JCo extract treatment (Figure 4a). Cyclin D1 interacts with CKD4 to modulate the cell cycle distribution of the G_1_ phase, and protein expression of both genes was downregulated by JCo extract in a time‐dependent manner. The data show that JCo extract leads to cell cycle arrest at the G_0_/G_1_ phase by regulating p53/p21 and CDK4/cyclin D1 protein expression.

To elucidate the molecular interactions underlying JCo extract induced apoptosis, the expression levels of apoptosis‐associated proteins from both pathways were measured. In the extrinsic pathway, the protein expression of Fas was increased, but the levels of Fas ligand (FasL) and Pro‐Caspase 8 were decreased; in the intrinsic pathway, protein expression of Bcl‐2 and Pro‐Caspase 9 was decreased, but Bax and cleaved Caspase‐9 were increased (Figure 4b). The expression levels of Pro‐Caspase 3 were shown to decrease in JCo‐treated cells, and activation of Pro‐Caspase 3 was blocked by a Caspase 3 inhibitor (Figure 4b,c). The data indicate that JCo extract triggers extrinsic and intrinsic apoptosis pathways and activates caspase cascade signaling to induce cell apoptosis.

### 3.5 Translocation of cell cycle regulators and caspase proteins in JCo extract treated cells

**FIGURE 5:**
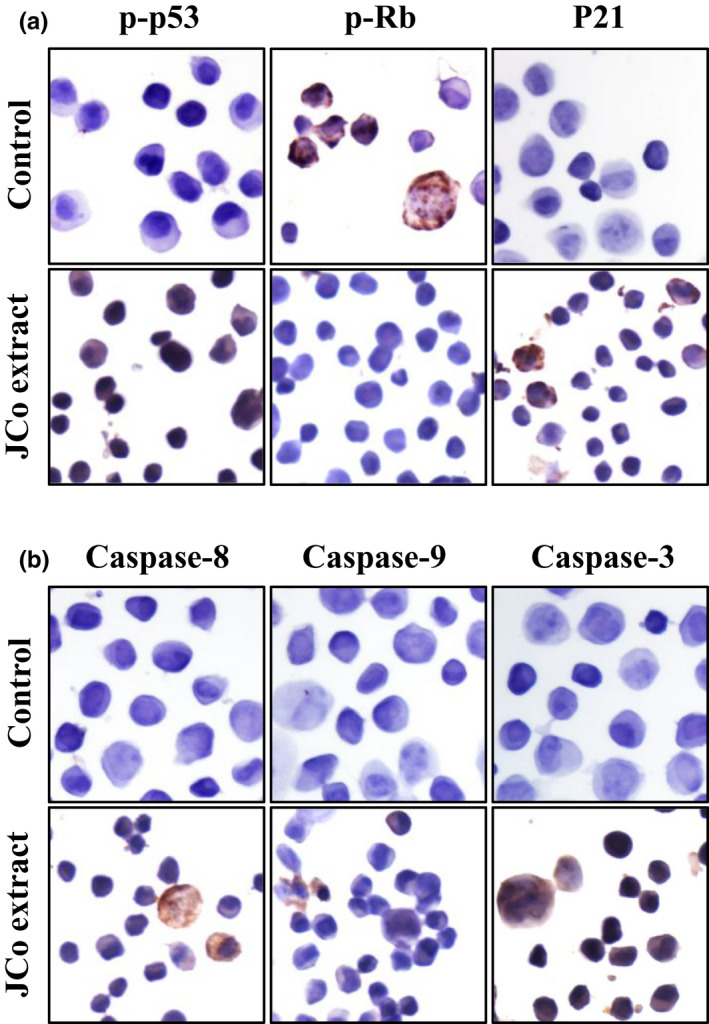
JCo extract activated cell cycle regulators and caspase proteins in esophageal cancer cells. CE81T/VGH cells were treated with JCo extract for 24 hr and the expression and localization of proteins, such as (a) cell cycle proteins (p‐p53, p‐Rb, and p21) and (b) apoptosis proteins (Caspase 8, Caspase 9, and Caspase 3), was shown via immunocytochemistry staining

CE81T/VGH cells were treated with JCo extract for 48 hr and protein expression and localization of cell cycle regulators and apoptosis‐related proteins was determined by immunocytochemistry staining. The p‐p53 and p21 proteins were increased and translocated into the nucleus, but p‐Rb was reduced (Figure 5a). The caspase cascade proteins, Caspase 3, 8, and 9, were enhanced and expressed in the nucleus (Figure 5b). In summary, JCo extract regulates the expression of cell cycle regulators and caspase proteins and triggers their translocation to induce cell cycle arrest and apoptosis.

### 3.6 Anti‐proliferative effect of JCo extract combined with 5‐FU

**FIGURE 6:**
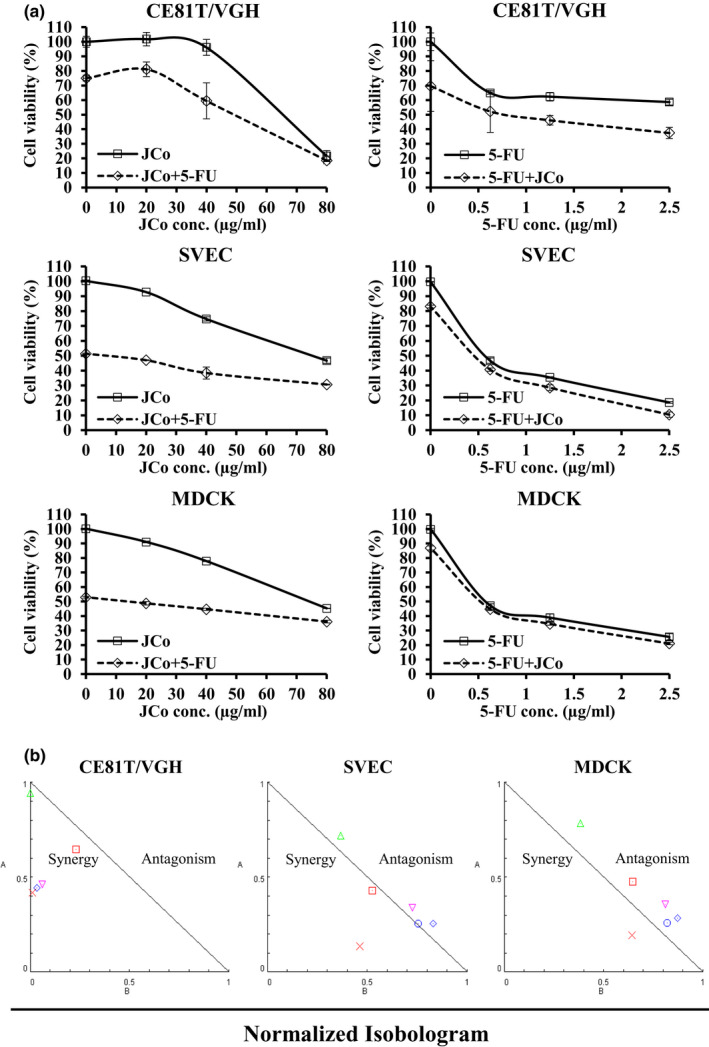
JCo extract enhanced 5‐FU induced cytotoxicity in esophageal cancer cells. Cells were treated with a combination of (a) JCo extract (20, 40, or 80 μg/ml) with or without 0.5 μg/ml 5‐FU; 5‐FU (0, 0.625, 1.25, 2.5 μg/ml) combined with or without 30 μg/mL JCo extract for 48 hr, and cell viability was measured via an MTT assay. (b) The results were analyzed using CompuSyn to calculate the normalized isobologram

To analyze the combined effect of JCo extract and 5‐FU, the CT81T/VGH cells were treated with a combination of JCo extract (0, 20, 40 and 80 μg/ml) and/or 0.5 μg/ml 5‐FU; 5‐FU (0, 0.625, 1.25 and 2.5 μg/ml) and/or 30 μg/ml JCo extract for 48 hr, and evaluated for cell viability by an MTT assay. The cell viability of the combination group was lower than that of JCo extract or 5‐FU only in ESCC cells, showed a synergistic effect determined by normalized isobologram (Figure 6). However, the effect was not obvious in normal cells SVEC and MDCK. These results indicate that JCo extract and 5‐FU together could have an enhanced anti‐cancer effect in ESCC cells.

## 4 DISCUSSION

Natural products from plants contain various components that are important in the development of new drugs for cancer, infection, and inflammation (Mann, 2002; Padumadasa et al., 2016; Rong et al., 2016; Soukand et al., 2015). Moreover, the therapeutic activity of natural products in traditional medicine and herbal medicine is attributed to functional group structures, especially polyphenolic compounds (Soobrattee et al., 2005). *Juniperus communis* (JCo) is a coniferous evergreen coniferous shrub that is widely spread throughout the Northern Hemisphere across the Himalayas from the Kumaun region (Khare, 2007; Moein et al., 2010; Nakanishi et al., 2004). *Juniperus communis* oil contains monoterpene hydrocarbons, such as α‐pinene, myrcene, sabinene, and β‐pinene (Bais et al., 2014; Cabral et al., 2012; Hajdari et al., 2015), most of which are polyphenolic compounds, indicating their anti‐cancer potential. Studies have shown that *Juniperus communis* L. berry water extracts induces p53‐associated cell apoptosis in human SH‐SY5Y neuroblastoma cells (Lantto et al., 2016), and increases the effect of 5‐FU in non‐small lung cancer A549 cells through regulation of AKT phosphorylation (Raasmaja et al., 2019). In addition, juniper berry oil from *Juniperus communis* is a chemo‐preventive dietary agent that inhibits cell proliferation and inflammation and induces apoptosis, resulting in suppression of colon tumor formation in azoxymethane‐treated rats (Yaman et al., 2019). In our previous study, JCo extract inhibited cell proliferation and induced cell cycle arrest and apoptosis in melanoma B16/F10 cells (Gao et al., 2019). In this study, we investigated the anti‐cancer potential of JCo extract in ESCC cells. The results demonstrate that JCo extract reduces cell viability, induces cell cycle arrest at the G0/G_1_ phase and cell apoptosis via regulation of relative protein expression, and has a synergistic effect when combined with 5‐FU in CE81T/VGH cells in vitro.

Esophageal cancer is one of the most frequently diagnosed cancers and the sixth leading cause of cancer‐related deaths worldwide, causing more than 400,000 deaths each year (Pennathur et al., 2013; Siegel et al., 2016). The overall 5‐year survival rate of late‐stage ESCC is less than 15%, and there has been little improvement in the past few decades, although therapeutic approaches have been developed (Liang et al., 2017). Presently, drugs from plants, regardless of whether they are crude extracts or isolated bioactive compounds, have received a lot of scientific attention in cancer therapy because of their higher selection for cancer cells, resulting in a lower possibility of side effects (Fulda, 2010; Wang et al., 2018). In this study, the IC_50_ values of JCo extract in ESCC cells were lower than those of normal cells after 24, 48, and 72 hr, indicating that JCo extract has higher selection for ESCC cells. However, in the 5‐FU treatment group, there was no obvious selection between ESCC and normal cells after 24 hr, and selection for ESCC was lower after 48 and 72 hr. These results suggest that JCo extract shows lower cytotoxicity to normal cells compared with 5‐FU, which reduces the possibility of causing side effects.

The cell cycle progresses from quiescence (G_0_ phase) to proliferation (G_1_, S, G_2_, and M phases), and then returns to a quiescent state. It is well known that cell cycle arrest in response to DNA damage or cell stress is essential to maintaining genomic integrity. Cell cycle checkpoints play a crucial role in controlling the inhibition of cell cycle transitions or induction of signal transduction after cell stress (Flatt & Pietenpol, 2000). The analysis of cell cycle distribution revealed that ECSS cells treated with JCo extract were blocked in the G_0_/G_1_ phase of the cell cycle, but there was an increase in the subG_1_ population. These results indicate that JCo extract inhibits cell proliferation through G_0_/G_1_ phase arrest in ESCC cells. Cyclin‐dependent kinases (CDKs) and their activated cyclins play key roles in mammalian cell cycle regulation, and CDK/cyclin complex activity depends on the balance between cyclins and cyclin‐dependent kinase inhibitors (CKIs). CKIs included p27 (kip1) and p21 (cip1), and their expression is regulated by the p53 tumor suppressor protein (Coqueret, 2003). In the cell cycle, it has been determined that CDK4/cyclin D and CDK2/cyclin E promote the passage of cells through the G_1_ and S phases, while CDK1/cyclin B regulates the transition of cells through late G_2_ and mitosis (Morgan, 1995). The phosphorylation of Rb is responsible for the transitions between G_0_/G_1_ and G_1_/S (Taya, 1997). In this study, JCo extract upregulated p53, p‐p53, and p21, and downregulated Rb, p‐Rb, CDK2, CDK4, cyclin A, cyclin B1, and cyclin D1, suggesting that JCo extract treatment blocked the cell cycle via regulation of cell cycle relative proteins, especially in the G_0_/G_1_ phase.

Apoptosis is a basic process that is crucial for the development and maintenance of cellular homeostasis within tissues. Apoptosis is a form of cell death with distinct morphological features, such as accumulation of subG_1_ phase cells, anoikis, chromatin condensation, DNA fragmentation, and apoptotic bodies, and it is triggered by specific signaling pathways (Chen et al., 2018). The changes in cell morphology may be an indicator of cell death and a reliable basis for identifying the presence of apoptosis. The a TUNEL assay of JCo extract‐treated cells gave TUNEL‐positive results for anoikis morphology, chromatin condensation, DNA fragmentation, and apoptotic bodies, indicating cells were undergoing apoptosis. Apoptosis signaling cascades are divided into two major pathways: the death‐receptor (Fas/FasL/Caspase‐8) and mitochondria‐mediated (Bax/Bcl‐2/Caspase‐9) pathways (Pfeffer & Singh, 2018). Both the death‐receptor and mitochondria‐mediated cell apoptosis‐associated proteins were regulated by JCo extract treatment. In addition, JCo extract induced activation of Caspase‐3 was blocked by the Z‐DEVD‐FMK Caspase‐3/7 inhibitor. These results indicate that JCo extract induces apoptosis in ESCC CE81T/VGH cells through the extrinsic and intrinsic apoptosis pathways.

5‐fluorouracil (5‐FU) was the first‐line treatment for ESCC therapy for several decades (Longley et al., 2003), but single agent chemotherapy of 5‐FU is no longer appropriate for ESCC therapy because of its toxicity, drug resistance, and side effects. Therefore, the development of combination therapies with other drugs, such as irinotecan and oxaliplatin, is used to improve prognoses and reduce the side effects of 5‐FU (Ferte et al., 2011; Wainberg et al., 2011). Combination therapy enhances the response rate and survival rate, but the side effects and drug resistance are still unknown. In this study, we combined JCo extract with 5‐FU for the treatment of ESCC cells. The results indicate that combination treatment causes more cell inhibition than JCo extract or 5‐FU alone, suggesting that JCo extract combined with 5‐FU synergistically inhibits ESCC cell growth. These studies indicate that JCo extract has great potential for the development of anti‐cancer agents or adjuvants for the combination therapy of ESCC in the future.

In conclusion, JCo extract inhibits the growth of ESCC cells by stimulating G0/G_1_ arrest via regulation of cycle regulatory proteins, such as p53, p21, CKD4, and cyclin D1. Moreover, JCo extract triggers cell apoptosis and apoptotic morphology, including anoikis, chromatin condensation, DNA fragmentation, and apoptotic bodies, in ESCC cells through intrinsic and extrinsic apoptosis pathways. JCo extract enhances the anti‐cancer effect of the clinical drug 5‐FU, suggesting that it can complement current chemotherapeutic treatment.

## CONFLICT OF INTEREST

The authors declare no conflict of interest.

## ETHICAL APPROVAL

This study does not involve any human or animal testing.

## Data Availability

The data that support the findings of this study are available from the corresponding author by reasonable request.
